# Evaluation of Dust Deposition on Parabolic Trough Collectors in the Visible and Infrared Spectrum

**DOI:** 10.3390/s20216249

**Published:** 2020-11-02

**Authors:** Rubén Usamentiaga, Alberto Fernández, Juan Luis Carús

**Affiliations:** 1 Department of Computer Science and Engineering, University of Oviedo, 33204 Gijón, Asturias, Spain; 2 Grupo TSK, C/Ada Byron, 220, 33203 Gijón, Asturias, Spain; alberto.fernandez@grupotsk.com (A.F.); juanluis.carus@grupotsk.com (J.L.C.)

**Keywords:** solar energy, dust reposition, parabolic trough collectors, infrared thermography

## Abstract

Solar energy is mostly harnessed in arid areas where a high concentration of atmospheric dust represents a major environmental degradation factor. Gravitationally settled particles and other solid particles on the surface of the photovoltaic panels or thermal collectors greatly reduce the absorbed solar energy. Therefore, frequent cleaning schedules are required, consuming high quantities of water in regions where water precipitation is rare. The efficiency of this cleaning maintenance is greatly improved when methods to estimate the degree of cleanness are introduced. This work focuses on the need for better detecting the degradation created by dust deposition, considering experimental data based on different air pollutants, and analyzing the resulting thermal and visible signatures under different operating environments. Experiments are performed using six different types of pollutants applied to the surface of parabolic trough collectors while varying the pollutant density. The resulting reflectivity in the visible and infrared spectrum is calculated and compared. Results indicate that the pollutants can be distinguished, although the reflectivity greatly depends on the combination of the particle size of the pollutant and the applied amount, with greater impact from pollutants with small particles.

## 1. Introduction

In recent decades, the harvest of solar energy based on photovoltaic and photothermal systems has experienced exponential growth [1]. The efficiency of the associated technology has greatly increased, while the cost has fallen substantially. Solar energy contributes to sustainable development, as it is an essentially inexhaustible energy resource that is clean and inexpensive [2]. In these conditions, the process of harnessing solar thermal energy to generate thermal or electrical energy is evolving very quickly; it is becoming a highly competitive field in which better quality and more efficiency are in constant demand [3].

A major environmental degradation factor in photovoltaic panels and solar thermal collectors is the concentration of atmospheric dust [4,5,6]. In the case of photovoltaic panels, the accumulation of dust due to gravitational settling and the presence of other solid particles on the surface greatly increases the reflection, which contributes to a considerable reduction of absorbed solar energy. This leads to a remarkable energy generation reduction, and thus important negative economic impacts. In the case of solar thermal collectors, the generated thermal power is also greatly affected, as the optical properties of the collectors are related to solar absorption. The reflectivity of the reflectors used in some types of collectors and the transmissivity of the glass covers can be significantly reduced when the dust is deposited on the surface. Photovoltaic panels with dirt, dust or bird droppings can significantly reduce its energy production capacity that can even lead to a loss of 15% of its capacity [7]. In the case of solar thermal collectors, dust deposition can even reduce the average performance by 60% [8].

The rain and the wind can help clean the surface of the panels and reflectors, but only partially. Therefore, different cleaning schedules are considered to remove the effect of dust on the performance of the systems [9]. The most common procedure uses around 0.5 L of water per square meter, which represents a very significant water consumption, especially for arid regions where water precipitation is rare. Moreover, in these desert areas, frequent dust storms increase dust deposition, which requires even more frequent cleaning schedules. When considering the economic impact of dust deposition, the cost of the cleaning operation must be added to the cost of lost energy [10]. Therefore, designing an efficient cleaning procedure is of utmost importance. The goal is to design cleaning schedules that minimize cleaning cycles while maintaining system performance. However, the cleaning schedules are dependent on many variables, such as weather, installation site or wind patterns. Thus, it is difficult to estimate the degree of cleanness. The efficiency of the designed procedures can be greatly improved when automated methods to estimate the degree of cleanness are introduced.

Dust detection methods can be based on the generated output energy from the panels [11]. For example, in ref. [12], an automated cleaning system is triggered when solar radiation is detected and the power output resulting from the radiation is less than 20% below the average that the system interprets as normal. This approach is a rough approximation of dust detection, as the resulting power greatly depends on other parameters, such as the amount of solar radiation or the angle between the incident ray and the panel. Another approach used in ref. [13] is based on specific dust sensors. This type of sensor includes a heater that creates an upward current of air. Then, another sensor component counts the number of particles with a size of over one micrometer. This sensor can detect dust, but only on very limited areas of the panel. A different approach is based on cameras used either in the visible or in the infrared spectrum [14]. In ref. [15], a method is proposed based on high-resolution color cameras where features are extracted from the hue layer of the HSV color space using the gray level co-occurrence matrix. Limited testing is provided, but the goal is to apply the method to images acquired from drones that scan an area that is covered by hundreds of panels in just a few minutes. Other similar methods based on visible cameras can be found in ref. [16,17,18]. The method presented in ref. [19] is based on infrared thermography, where the dust is detected based on temperature differences. In ref. [20], RGB and infrared images are also used for soiling detection, but only using a visual qualitative analysis. Infrared thermography is a mature and accepted technology widely used in the inspection, monitoring and fault diagnosis of photovoltaic installations. Methods based on infrared thermography are commonly used to detect defects in photovoltaic panels [21,22,23,24] and, in general, in electric installations [25,26,27], but research on dust detection is very limited.

This work evaluates the capability of infrared thermography for the detection and assessment of dust depositions on parabolic trough collectors (PTCs). The work is complemented with an analysis of images acquired in the visible spectrum from the reflection of laser light on the surface of the reflectors. The reflectivity in both the visible and the infrared spectrum is calculated and a model is trained to evaluate the feasibility of automated classification.

Experiments are performed using a reliable and robust method for polluting the reflectors. The method seeks to create polluted samples under controlled and repeatable conditions. For this, a procedure that does not require special materials has been developed. Six types of pollutants have been selected to be representative of polluted reflectors: ash, red soil, cement, limestone, coarse sand and fine sand. Different quantities of pollutants are mixed with water and poured onto the panels into cylinders that prevent the water from leaking out. Subsequently, the water is evaporated with a drying process and the cylinders are removed, leaving the pollutant on the reflector. Following this procedure, four reflectors have been polluted with different quantities: 5 g, 2 g, 0.5 g and 0.1 g (pollutant density from 0.1 to 0.001 g/cm${}^{2}$)—in all cases, on an area of 50 cm${}^{2}$. Therefore, areas of polluted reflectors with different densities have been created. Reflectivity in the visible and infrared spectrum is analyzed.

The remainder of this paper is organized as follows. Section 2 introduces thermosolar energy and describes PTCs. Section 3 briefly reviews the foundations of infrared thermography and the estimation of reflectivity. Section 4 presents the experimental procedure and the materials and pollutants tested. Section 5 discusses the results obtained. The conclusions are reported in Section 6.

## 2. Parabolic Trough Collectors

Solar thermal, or thermosolar, energy is based on harnessing the energy of the sun to produce heat. The heat generated can be used for various purposes such as the generation of hot water. However, in industry, heat is generally used for the generation of electrical power.

Both solar thermal energy and photovoltaic energy are used to generate electricity. In general, generating electricity using photovoltaic energy is more affordable. However, because the process of harvesting photovoltaic solar energy directly converts sunlight into electricity, energy can only be produced when the sun is shining, making it complex and expensive to store in batteries. Therefore, the photovoltaic energy harvesting process cannot produce electricity at night; unfortunately, the first hours of the night (between 6 p.m. and 11 p.m.) is the period when most energy is consumed. On the other hand, solar thermal energy can be stored more easily in molten salts, thus that stored energy can be used when it is needed, regardless of whether the sunlight is shining at that time. Currently, and due to the advantages and disadvantages of both forms of power generation, the most innovative power generation plants are opting for a hybrid approach which combines both solar thermal energy and photovoltaic energy. This way, energy can be stored when there is sunlight and, potentially, it is possible to generate electricity during all 24 h of the day.

Parabolic trough collectors (PTCs) are among the various alternatives for the generation of solar thermal energy [28]. A PTC consists of a highly reflective panel constructed of silver-plated glass. The panel is shaped like a parabola and redirects the radiation of the sun into a concentrator made up of a pipe covered with borosilicate glass. The pipe contains low-viscosity oil with a high boiling point that absorbs the concentrated solar energy from the tube wall [29]. The oil concentrates solar radiation and heats up. This heat is subsequently transformed into electrical energy by a heat exchanger that generates steam to power a turbine. Subsequently, a cooling tower returns the water to the liquid state to restart the process again. To maximize sunlight harvesting efficiency, the pipe is placed along the focal line of the PTC and the position of the sun is tracked for normal incidence of solar radiations [30]. PTCs are a proven and reliable technology that presents a far more cost-efficient solution than other solar power technologies [31].

**Figure 1:**
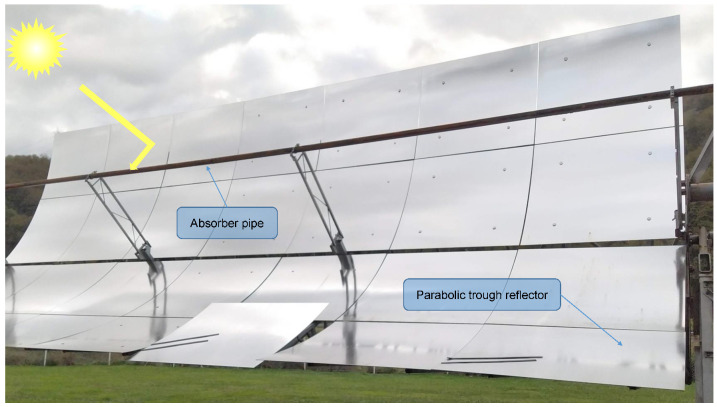
Parabolic trough collector.

Figure 1 shows an image of a PTC used to generate electricity from sunlight. Commercial plants use a very large number of PTCs that can generate hundreds of megawatts. For example, the concentrated solar power plant SEGS in California has around 1 million PCTs and generates 354 megawatts [32].

## 3. Infrared Thermography

Infrared thermography is based on the measurement of infrared radiation emitted by all objects with a temperature greater than 0 K [33]. The intensity of the radiation is related to the temperature of the object; therefore, with infrared thermography, the temperature of an object can be estimated at a safe distance based on the radiant energy [34]. In addition, it enables two-dimensional viewing, making thermographic images easy to interpret [35].

Radiant energy is dissipated due to absorption, transmission and reflection. The fractions of total radiant energy dissipated due to reflection is called reflectivity. Body absorption and transmissivity are the equivalents of transmission and absorption. The spectral absorption, $\alpha_{\lambda }$ (ratio of spectral radiant power absorbed by the object), the spectral reflectance, $\rho_{\lambda }$ (ratio of spectral radiant power reflected by the object) and the spectral transmittance, τλ (ratio of spectral radiant power transmitted by the object are the parameters used to describe these phenomena. The sum of these three parameters must be 1 at any wavelength, as shown in Equation (1). As can be seen, these three parameters depend on the wavelength, λ.

(1) $$\alpha_{\lambda }+\rho_{\lambda }+\tau_{\lambda }=1$$

In the case of opaque materials, Equation (1) becomes Equation (2), since the transmittance is null.

(2) $$\alpha_{\lambda }=1-\rho_{\lambda }$$

Black bodies are materials where the transmissivity and reflectivity are zero. These materials absorb all the radiant energy ($\alpha_{\lambda }=1$).

The Stefan–Boltzmann law Equation (3) describes the radiation emitted by an object, *W*. This radiation depends on temperature (*T*), but also on the emissivity of the object ($\varepsilon_{\lambda }$). The emissivity is formally defined as the ratio between the radiant energy emitted by the body and the radiation that a black body would emit at the same temperature. The value of σ is constant.

(3) $$W=\varepsilon_{\lambda }\;\cdot \;\sigma \;\cdot \;T^{4}$$

When there is no transmission or reflection, i.e., all the radiation energy that an object receives is absorbed, the absorption capacity is 1. At a constant temperature, all the absorbed energy must be re-radiated (emitted) so that the emissivity of said body is 1. Thus, the absorption capacity in a black body is equal to the emissivity, which is 1. According to Kirchhoff’s law, the emissivity and the absorption capacity of any material are equal for any temperature and wavelength. This can be expressed as Equation (4).

(4) $$\varepsilon_{\lambda }=\alpha_{\lambda }$$

From Equations (2) and (4), Equation (5) is obtained for opaque materials.

(5) $$\rho_{\lambda }=1-\varepsilon_{\lambda }$$

Emissivity and reflectivity are formally defined for a wavelength λ. If the emissivity is constant and independent of the wavelength, the Stefan–Boltzmann law is expressed as Equation (6). This type of body is often called a gray body.

(6) $$W=\varepsilon \;\cdot \;\sigma \;\cdot \;T^{4}$$

Substituting Equation (5) into Equation (6) gives Equation (7). Solving this equation for ρ results in Equation (8).

(7) $$W=\left(1-\rho \right)\;\cdot \;\sigma \;\cdot \;T^{4}$$

(8) $$\rho =1-\frac{W}{\sigma \;\cdot \;T^{4}}$$

The reflectivity of an object can be calculated using Equation (8) based on the measured radiation when the temperature is known. In this way, infrared thermography can be used to measure the reflectivity of objects. This method needs to know the temperature of the object. There are two ways to measure this parameter. First, the object of study can have a built-in thermocouple that provides its temperature by means of a contact measurement. Second, the temperature of the object can be measured as long as there is a part of the object at the same temperature where the surface has a known emissivity. In this second option, it is usual to paint a part of the object to achieve a high and known emissivity in that area. The painted area is the reference area that is contrasted with the rest of the material in order to measure its reflectivity. Another alternative to this second option is to measure the emissivity of the object’s surface, although this approach presents problems when the emissivity is low.

## 4. Materials and Methods

### 4.1. Reflectors and Pollutants

To determine the feasibility of detecting dust in PTCs, a controlled experiment is proposed where, in a systematic way, a certain level of contamination is applied to a set of test panels.

**Figure 2:**
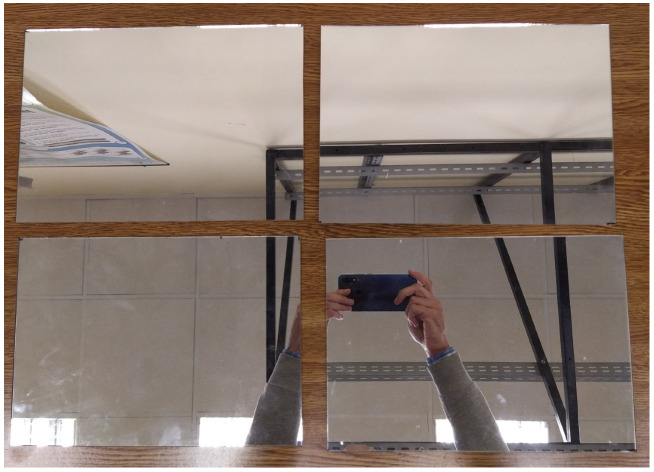
Reflectors used for the experiments.

For conducting experiments, five pieces of the reflectors used in the fabrication of PTC are used. The test reflectors are made of silver-plated glass with high reflectivity, since the objective is to reflect the light toward the collector where heat is generated. The reflectors have a thickness of 4 mm and a dimension of 29.5 × 20.5 cm. The only difference compared to a reflector used in the collectors is that they are not curved, as they appear in parabolic collectors. Eliminating the curvature makes testing easier without modifying its properties. Figure 2 shows an image of some of these panels where their high reflectivity can be clearly seen.

**Figure 3:**
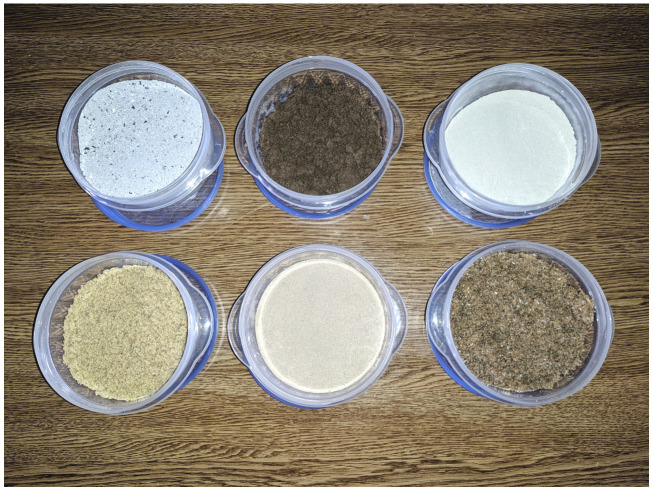
Pollutants used for the experiments. From left to right and from top to bottom: ash, red soil, cement, limestone, fine sand and coarse sand.

Six types of pollutants with different physical characteristics have been considered for the experiments. The pollutants are representative of real contamination on the reflectors on different conditions and real installations. The pollutants are shown in Figure 3. The pollutants are the following:

- Ash. This carbon-based pollutant is an example of incomplete combustion; it is emitted by thermal power plants and in the exhaust of vehicles.
- Red soil. Red soil is dry earth that is set in motion by the wind and can later be deposited on the panels. This type of soil can be found in warm climates and contains inorganic and organic minerals.
- Cement. Cement is an example of a powder with organic and inorganic salts that is soluble and that adheres to the glass surface.
- Limestone. Limestone is a pollutant that consists mainly of calcium carbonate and is frequently used in buildings.
- Fine sand. Most solar thermal stations are installed in places with a great amount of solar radiation, such as deserts. In these facilities, the most common dust that contaminates the facilities contains sand similar to this type. This sand used for testing was obtained from the Kuwait desert [36].
- Coarse sand. A second type of sand obtained from a beach in the north of Spain was used. This type of sand is characterized by a much larger grain size and, therefore, its behavior is expected to be different from that of fine sand.

Table 1 shows the particle size for each of the considered pollutants. As can be seen, there are significant differences in particle size, with the coarse sand being the largest, as it contains eroded particles from the shells of marine animals.

The types of pollutants used in the experiments are consistent with previous works [7,37]. They are considered representative of different natural conditions occurring in industrial installations. Ash and limestone are pollutants that affect the reflectors when they are installed close to urban areas. They originated in thermal power stations, vehicles and buildings. Red soil results from the wind moving dry terrain in some deserts. Sand is the most common pollutant in desert areas where solar energy is commonly harvested. Not all deserts have the same type of sand; this is why two types of sand are used in this work. Cement is a material with small particles representative of contaminants in rain. Therefore, they represent a valid and complete experimental plan to evaluate the dust estimation.

### 4.2. Cameras

The infrared camera used in the experiments is a Flir T450sc. The camera has an 18mm lens and a sensor resolution of 320 × 240 resolution. The temperature range is configurable within several available ranges. In the experiments, the range [−20, 120 ${}^{{}^{\circ}}$C] was selected. The manufacturer reports a measurement precision of ±1 K and a sensitivity of less than 30 mK at 30 ${}^{{}^{\circ}}$C. The longwave infrared camera operates at a wavelength between 7.5 and 13 μm. The type of detector used in the camera is an uncooled microbolometer. Full technical specifications are shown in Table 2.

For the infrared emissivity and reflectivity measurement experiments, Scotch^TM^ Premium Vinyl Electrical Tape 88 3M was used. This type of tape has a known emissivity (0.96) and it is commonly used as a reference material in experiments with infrared cameras.

The emissivity of the reflector was measured using the reference emissivity material method [38]. According to this method, the object on which the emissivity is to be measured is heated with a piece of electric tape with known emissivity glued to the surface. The reference temperature on the electrical tape is used to measure the emissivity of the panel.

To estimate the reflectivity of the panels with the pollutant, a heating process was carried out at a temperature close to 50 ${}^{{}^{\circ}}$C. This temperature was more than double the ambient temperature inside the room (20 ${}^{{}^{\circ}}$C). The increase in temperature was used to distinguish the object under study—in this case, to distinguish the reflectors from the environment. In this way, the influence of the reflected temperature, the resulting temperature as a result of the reflection of the environment on the object, was lower. Therefore, the heating of the piece made it easier to analyze the areas with pollutants on the panel.

**Figure 4:**
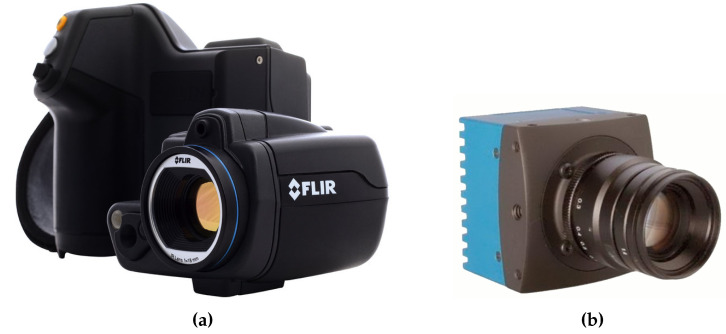
Cameras used in the experiments. (**a**) FLIR T450sc, (**b**) Mikrotron EoSens MC1364.

In order to analyze the properties of the reflectors in the visible spectrum, a color camera and a laser projector were used. The camera used is the Mikrotron EoSens MC1364, with the technical specifications indicated by the Table 3. The laser projector is the Logitech R400 pointer. Figure 4 shows the cameras used.

**Figure 5:**
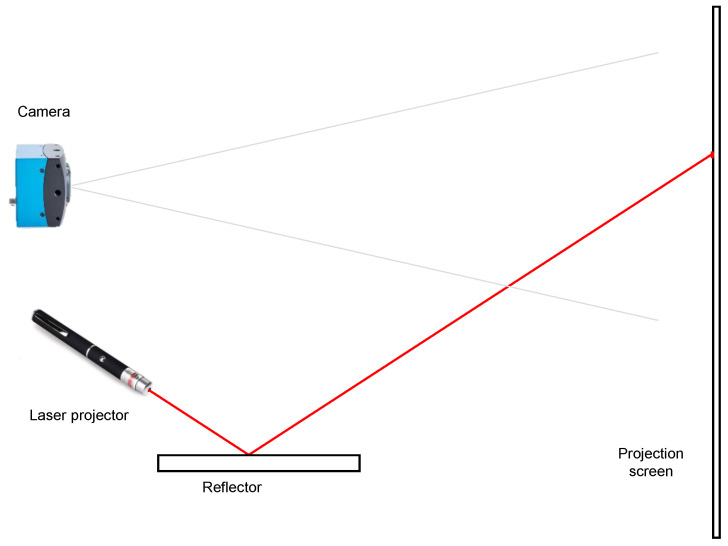
Measurement of influence of the pollutants in the visible spectrum.

The procedure for the measurement of the influence of the pollutants in the visible spectrum is shown in Figure 5. The laser projector was focused on a certain part of the glass panel. The reflection of the laser on a projection screen was observed by the camera. Given the field of view of the camera, the laser could be focused on different areas of the panel, contaminated or clean, to determine the degree to which they reflect light. The distance between the panel and the projection screen was approximately equal to the distance in a real solar thermal panel between the reflector and the absorber pipe where the heat accumulated to be later transformed into electrical energy.

The proposed approach to measure the reflectivity in the visible spectrum is similar to other available sensor configurations [8].

### 4.3. Applying the Pollutants to the Reflectors

Before starting the experiments to apply the pollutants to the reflectors, a drying process was carried out. For several hours, each pollutant was placed on heating plates with the aim of eliminating its moisture. The pollutants were then left in a room with constant humidity and temperature conditions for one week. This process was followed to ensure that all pollutants had similar starting conditions.

The next step was the weighing of the pollutant. To create areas with controlled dust, a certain amount of pollutant was weighed before the artificial application onto the surface of the reflector.

**Figure 6:**
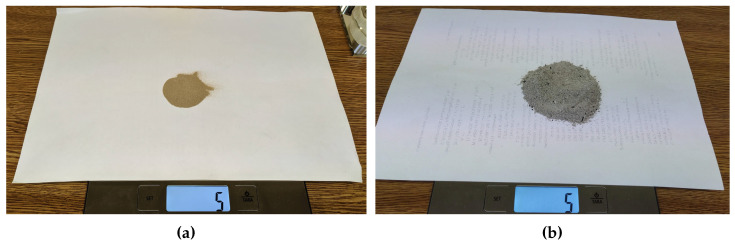
Weighing of the pollutants. (**a**) Fine sand, (**b**) ash. In both cases, the pollutant weighs 5 g.

The density of the pollutants varied. This caused—for example, in the case of ash—a certain weight to represent a greater volume than with other pollutants with higher density, as can be seen in Figure 6.

In order to apply the pollutants to the reflectors in a controlled and reproducible way, a specific procedure was proposed. A set of six cylinders were placed on the reflectors: one cylinder per pollutant. The cylinders were glued to the reflector using elastic clay. The material used to glue the cylinders to the panel prevented the water, which was used to apply the pollutant uniformly, from escaping the cylinders. In addition, the bonding material was easy to remove and reuse. The cylinders used in the experiments were 8 cm in diameter (50 cm${}^{2}$).

The weighed pollutant was then diluted in 0.2 L of hot water. The goal was to achieve uniform contamination within a given surface. Once the hot water was mixed correctly, it was poured into each of the cylinders. Each diluted pollutant was poured into one of six cylinders, one per pollutant. No mixtures of different pollutants were applied. This enabled testing the effect of each pollutant on the reflector independently. In a real installation, pollutants can be mixed but are predominantly of one type, such as the type of sand of a particular desert.

The reflectors were then subjected to a hot air drying process; the objective of the drying process was to cause the evaporation of the water. In this way, the reflector was contaminated with a controlled amount of pollutant. The drying process was applied for 12 h. Once the water evaporated, the cylinders were carefully removed. Finally, the reflector contaminated with a specific amount of pollutant was obtained—that is, with a specific density of contamination.

The same process was repeated varying the amount of pollutant used. Four variations were considered with different quantities: 5 g, 2 g, 0.5 g and 0.1 g. Based on these quantities, and considering the size of the cylinders where the pollutant is poured, the resulting pollutant density can be seen in Table 4.

## 5. Results and Discussion

### 5.1. Polluted Reflectors

**Figure 7:**
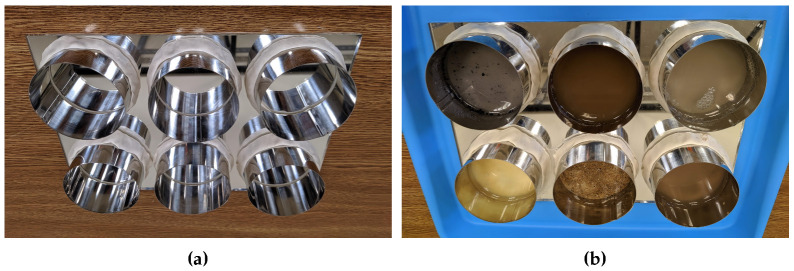

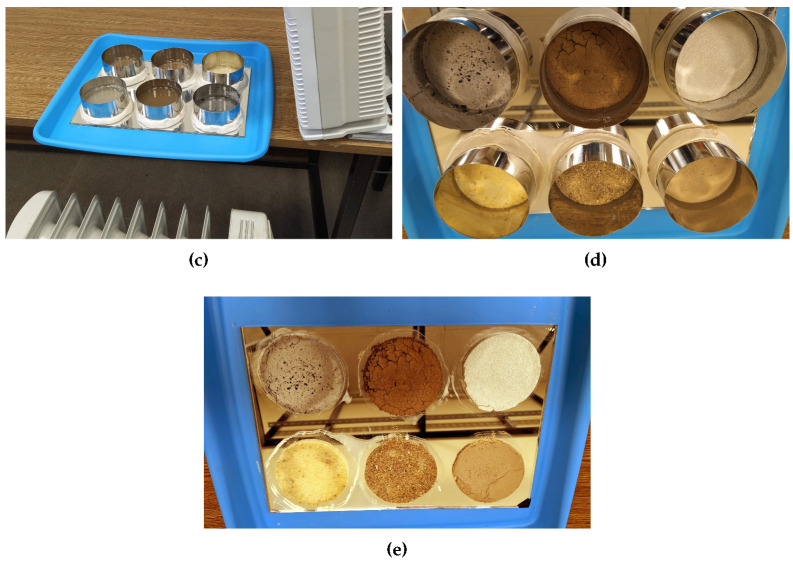
Results of the procedure for the application of 5 g of pollutants on the reflectors. (**a**) Cylinders glued to the reflector using elastic clay; (**b**) diluted pollutant poured in the cylinders; (**c**) drying of the diluted pollutant; (**d**) result after the drying procedure; (**e**) result after removing the cylinder.

Figure 7a shows the cylinders placed on the reflector. These cylinders were glued to the reflector using elastic clay. The diluted pollutants were poured into the cylinders. The result is shown in Figure 7b. Figure 7c shows the drying process to achieve the evaporation of the water. In Figure 7d, it can be seen that the water evaporated and only the pollutant remained on the glass surface. Figure 7e shows the final result. This example shows the result of application of 5 g of pollutant per cylinder. As can be seen, the pollution of the reflector was achieved in a uniform way by applying a controlled amount of pollutant.

**Figure 8:**
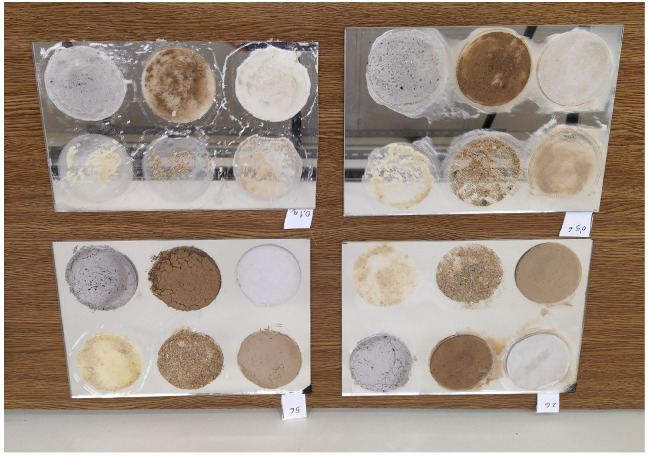
Reflectors polluted with different densities. From left to right and from top to bottom: 0.1 g, 0.5 g, 5 g, 2 g. All pollutants were applied in the same order (ash, red soil, cement, limestone, coarse sand and fine sand) except the reflector with 2 g, where the first and second row were swapped.

Figure 8 shows the final result after the application of the pollutants with the four considered densities. These are the reflectors that were used for the experiments. It can be seen how the pollutants with a smaller particle size covered the polluted area much better for the same quantity. This effect was more visible as the amount of pollutant was reduced. In these cases, the pollutant with large particles was concentrated in some areas, where the rest of the reflector was left similar to non-polluted areas.

### 5.2. Results in the Infrared Spectrum

**Figure 9:**
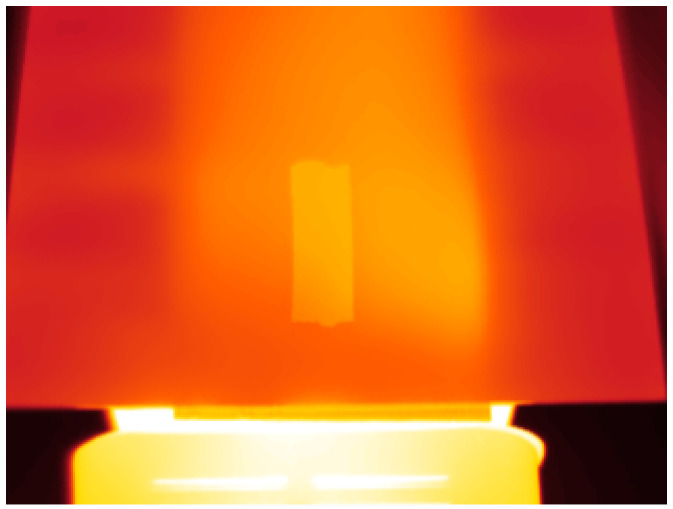
Infrared image of the reflector with electrical tape on the surface.

Figure 9 shows an infrared image acquired during the experiment to measure the emissivity. As can be seen, the area with the electrical tape showed higher radiation as it had a higher emissivity value than that of the reflector surface.

The result of the emissivity measurement indicated that the surface of the reflector had an emissivity of 0.854 with the standard deviation 0.010. Therefore, its reflectivity was 0.146 for the wavelength of the camera, from 7.5 to 13 μm. This information can be used to estimate the temperature on the surface of the reflector and use it as a reference for the estimation of the reflectivity, following the procedure previously described.

Infrared thermographic measurement includes uncertainties that are generally difficult to discern, as every single standard uncertainty of influencing variable need to be determined either through type A or type B analysis. Moreover, correlation between governing variables should be established and some components need to be determined using numerical Monte Carlo simulations [39]. In infrared cameras, the estimation of the emissivity and reflectivity also depends on internal calibration parameters not exposed to the user and only available to software tools developed by the manufacturer, such as the RBFO parameters. In this work, the variability of the measurements was analyzed using confidence intervals.

In order to calculate a confidence interval for the mean reflectivity of the panel, the first step is to check whether the data are normally distributed. The hypothesis of normality performed using the Shapiro–Wilk test concludes the null hypothesis cannot be rejected (*p*-value = 0.114). Hence, the data is normal. Therefore, the mean reflectivity of the panel is 0.146 ± 0.013 at 95% confidence level (calculated assuming the Student’s *t*-distribution).

**Figure 10:**
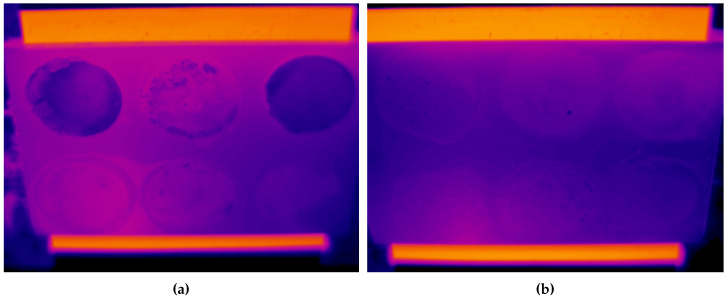
Infrared images acquired for the evaluation of dust detection. (**a**) Reflector polluted with 5 g; (**b**) reflector polluted with 0.5 g. From left to right and from top to bottom: ash, red soil, cement, limestone, coarse sand and fine sand.

Figure 10 shows two infrared images of two reflectors polluted with different quantities, 5 g and 0.5 g. The hot area under the reflectors is a heater used to increase the temperature of the reflectors. A qualitative analysis of the images makes it possible to determine that the polluted areas are clearly distinguishable in the images. In addition, the images show that the thermal contrast between the polluted and the clean area was greater with more pollution.

The images also show how the thermal response of pollutants was not the same. The pollutants in the upper row (ash, red soil, and cement) show a higher thermal contrast. Thus, detecting these pollutants in the images is easier. On the other hand, the pollutants in the bottom row (limestone and sands) show a thermal response more similar to that of the reflector surface, so their identification is more complex.

For each of the four polluted reflectors with a different amount of contaminant (5 g, 2 g, 0.5 g, 0.1 g) and type of pollutant (ash, red soil, cement, limestone, fine sand and coarse sand), a calculation of its reflectivity in the infrared spectrum was performed. The reflectivity for the measurement wavelength, in the case of the camera used from 7.5 to 13 μm, was calculated using Equation (8), given the temperature of the object and the measurement of infrared radiation.

The heating of the polluted reflectors was not completely uniform due to the difficulty of applying the same heat to a large area. Therefore, to estimate reflectivity, an area of the clean reflector that is as close as possible to the analyzed pollutant was always used as a reference. In this way, it was determined that both zones (polluted and reference) had the same temperature and were comparable.

The average results of the reflectivity calculation and the variability are shown in Table 5. The resulting reflectivity was in many cases similar to that found in the clean reflector (0.146). However, there are cases where the reflectivity was higher than that of the reflector, particularly in the case of ash, red soil and cement. In these cases, a higher reflectivity than that of the clean reflector was obtained. These results indicate that in the infrared spectrum the reflectivity of the reflectors increases when they are polluted—that is, the behavior is opposite to that of the visible wavelength. This phenomenon can also be observed in the images shown in Figure 10. For the same temperature, polluted areas emitted less radiation (they can be seen darker)—that is, their emissivity was lower, which indicated a higher reflectivity according to the principles of infrared thermography.

**Figure 11:**
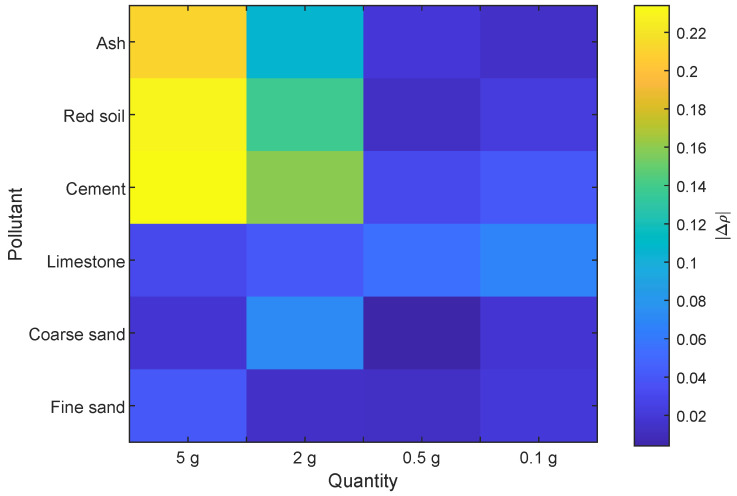
Reflectivity differences between polluted and clean reflector.

Figure 11 shows the differences in reflectivity differences between the polluted and clean areas of the reflector for each pollutant and quantity. In this color map, it can be clearly appreciated where the largest differences in reflectivity are found. The largest thermal contrast appears with the pollutants ash, red soil and cement. However, this difference decreases as the quantity of pollutant is reduced. For a concentration of 0.5 and 0.1 g the differences are similar to those of the rest of the pollutants.

For the pollutants limestone, coarse sand and fine sand, the detected differences in reflectivity were very low. The result was similar for cement, red soil and ash when the quantity was reduced to 0.5 g and less. In these cases, average differences even below 0.1 were obtained. In all cases, this variability was between 0.01 and 0.04, which indicated differences in reflectivity of 0.1 that are close to the statistical limit of significance. Confidence intervals around the average of these measurements overlap the confidence interval of the clean reflector. This indicates that, on average, these pollutants cannot be distinguished at a high confidence level. However, Even in these cases of low differences, the thermal contrast between polluted and clean areas is visible in the infrared image, as can be seen in Figure 10. Thus, a computational tool could actually identify pollutant even in small concentrations in the infrared images for the considered densities.

### 5.3. Results in the Visible Spectrum

To obtain the results in the visible spectrum, the procedure described has been followed with the projection of the laser and the measurement of the reflection using a camera. To obtain more robust results, multiple images have been taken, focusing the laser on different positions within each of the polluted areas.

**Figure 12:**
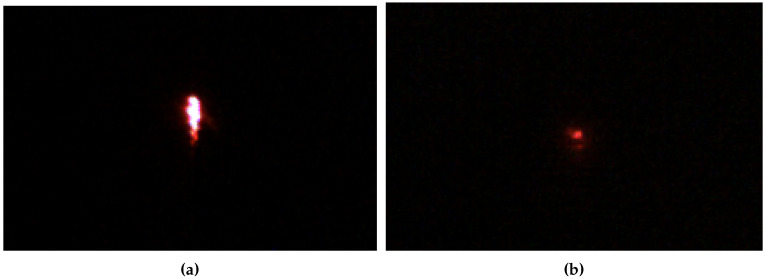
Visible images of the reflected laser. (**a**) Clean reflector, (**b**) Reflector polluted with 0.1 g fine sand.

For the experiments, the exposure time is fixed on the camera sensor and an aperture is configured in the optics so that only high intensity light is observed in the image, that is, laser light. The images resulting from the reflection of the laser on a clean reflector and one polluted (0.1 g of fine sand) are shown in Figure 12. As can be seen, the reflected area in the clean reflector is larger and with more intensity than in the polluted reflector.

The images obtained are transformed from RGB color to HSI space (hue, saturation, intensity). This color model allows the intensity of the light to be extracted regardless of its color, which in this case is red, the wavelength of the laser. The intensity channel is thresholded with a fixed value. Those pixels that have an intensity greater than 10% of the scale range are segmented. The resulting region is processed to measure its average light intensity. This process is carried out for all the images obtained with the reflection on a certain pollutant and the results are averaged. The result is an average intensity value. However, the absolute value is not of interest. The significant value is the relationship between the intensity obtained for a pollutant and the intensity on the clean reflector, that is, the relative reflectivity. A value of 100% means that the laser light is reflected the same as on a clean reflector, 50% means that only half of the light is reflected and 0% indicates that the camera is not able to observe the reflection.

The results of the relative reflectivity experiment in the visible spectrum are shown in the Table 6. The table shows that with a quantity of 5 g of pollutant the camera does not observe reflection, regardless of the pollutant. This is an extreme case where all the light is absorbed by the pollutant. For this quantity of pollutant, the panel stops fulfilling its function, as it is not able to redirect the light to the heat concentrator. Coarse sand is the only pollutant that produces reflection for a quantity of 2 g. The reason is the particle size of the pollutant. By having a larger particle size, it covers less area than the rest for a certain quantity. This causes the reflection in areas of the contaminated area to be visible by the camera, that is, the panel is capable of reflecting a part of the light. However, the results also indicate a large variability. Reflection greatly varies depending on the particular area the laser projector is focused. For a quantity of 0.5 g of coarse and fine sand and limestone reflect some of the light, but cement, red soil and ash still absorb all the light and do not generate reflection. Again, the phenomenon is related to the particle size and the area covered by the pollutant. Smaller particle size contaminants continue to cover the entire polluted area even for small quantities. For a quantity of 0.1 g all pollutants generate reflections except for cement. Cement dissolves very well with water and after pouring and drying it covers the contaminated area even for small quantities. The results also show that even small amounts of pollutant seriously affect the relative reflectivity, producing values on average below 50% in all cases. In general, it is observed that the reduction in reflectivity depends on the type of pollutant and its quantity per unit area.

#### Machine Learning Classification

The results presented in Table 6 indicate that, on average, a polluted reflector can be detected based on the relative reflectivity. As can be seen, in all cases the relative reflectivity percentage is always bellow 50% regardless pollutant type and density. Therefore, a naive binary classifier could be built based on this variable and threshold. However, single images are more difficult to distinguish. A machine learning model has been trained to evaluate the feasibility of dust detection using these images.

A deep neural network is used to analyze the images acquired in the visible spectrum. This type of neural network has been used recently to analyze and classify complex imagery with outstanding performance. Moreover, a pretrained network can be used as a starting point using transfer learning [40], greatly simplifying the complexity of the training procedure and requiring a smaller number of training images. A pre-trained model is trained with a very large dataset, and then only some of the final layers of the network need to be trained to finely tune the network for the specific dataset.

The model used in this work is the Inception-ResNet v2 architecture [41], which combines the Inception architecture with residual connections to achieve state of the art in terms of accuracy. This network has been trained with more than a million images from the ImageNet database. The network is 164 layers deep and includes rich feature representations for a wide range of images.

The selected architecture is designed for 1000 classes. Thus, the last two layers used for the final classification are replaced (fully connected and classification). In this work only two classes are used: clean and polluted. The weights of earlier layers in the network are locked by setting the learning rates to zero. Then the network is retrained using images acquired from clean and polluted reflectors with varying quantity and pollutant type. The dataset is composed of 100 images of each class, reserving 30% for testing. Moreover, the data set is augmented using translations and reflections. This augmentation increases the number of images in the dataset and prevents the network from overfitting.

**Figure 13:**
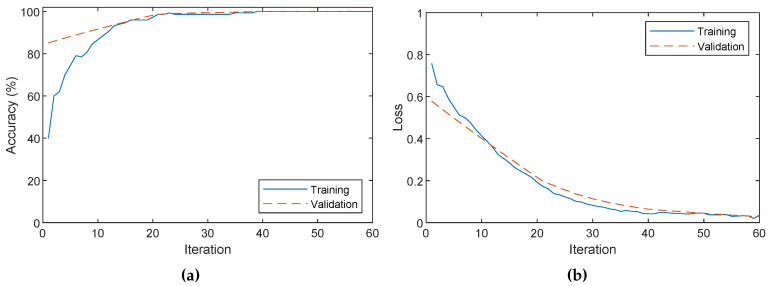
Result of the training of the deep neural model. (**a**) Accuracy, (**b**) loss.

Figure 13 shows the accuracy and loss during training. The network is trained using a learning rate of 0.0002, a momentum 0.9, a weight decay of 0.0001 and a batch size of 10. The networks converges quickly in 10 min with excellent performance. The resulting network is then applied to the test set, producing a 100% accuracy. This result indicates that single images acquired in the visible spectrum can be used accurately to detect dust on the reflectors, even with low quantities.

The machine learning classification in this work only performs very limited experiments. It is only applied to the visible spectrum because infrared images should be processed using a completely different approach. Visible images are obtained from the reflection of particular pollutants. Thus, one image correspond to either a clean or polluted reflector (with different type and concentration). Infrared images, on the other hand, as seen in Figure 10, show different areas of polluted reflector and clean reflector in the same image. Thus, a binary classifier cannot be applied to this type of image. The solution in this case would be a machine learning model that performs semantic segmentation, such as R-CNN [42]. However, this would require a large number of polluted panels with labeled images.

## 6. Conclusions

This work presents an evaluation on the feasibility of detecting dust on parabolic trough collectors using infrared thermography and compares the results with visible images. Considering the important impact that the deposition of dust and other pollutants has on the reflectors, it is of enormous interest to have methods that are automatically capable of detecting the problem in order to apply the appropriate measures. In remote areas where thousands of reflectors are typically installed, it is very complex and expensive to perform manual maintenance and inspection. Automation of dust detection and possible automated cleaning operations could provide a huge competitive advantage. In addition, studies that analyze the results of the visualization of the polluted reflectors in various light spectra help in the interpretation of the possible consequences.

The results in the infrared spectrum indicate that the pollutants can be distinguished from the clean reflector, therefore, their detection in the infrared is feasible. Thermal contrast due to differences in reflectivity depends on the combination of pollutant type and applied amount. The contrast is especially significant when the density of the pollutant is high (0.099 g/cm${}^{2}$ and 0.039 g/cm${}^{2}$) and particularly for contaminants with small particles (cement, red earth, and ash). In these cases, the polluted reflector has a higher infrared reflectivity than the clean panel, that is, the polluted reflector has a lower emissivity. The result of the experiment is that the apparent temperature of the polluted reflector is lower than that of the clean panel. As the amount of pollutant is reduced, the thermal contrast also decreases. This causes the signal to noise ratio to be low and therefore difficult to detect in general purpose applications. The results also indicate that, for the same amount of pollutant, the thermal effect varies depending on the type: those pollutants that contain smaller particles cause more variation in the thermal response. This type of pollutants is easier to detect in the infrared spectrum. Results indicate that all the considered pollutants (ash, red soil, cement, limestone, coarse sand and fine sand) with concentrations of 0.039 g/cm${}^{2}$ can be quantitatively distinguished in the infrared spectrum. Moreover, qualitative analysis on the infrared images also shows the polluted areas, making the detection of dust deposition possible.

The experiments have been complemented with reflectivity measurements in the visible spectrum. For this, a procedure has been designed based on a laser projection and a programmable camera with fixed exposure time. The results in the visible spectrum also indicate that pollutants with small particles have a greater impact on visible reflection. For the same amount of pollutant, the larger particles are concentrated in only one part of the analyzed area, thus allowing reflection on the rest. This causes some of the light to be reflected to the concentrator. On the other hand, those pollutants with a smaller particle size cover the polluted area better and reflect worse. Measurement results for reflectivity in the infrared spectrum are contrasted with reflectivity in the visible spectrum for various types of pollutants. When reflectivity increases in the infrared, it decreases in the visible spectrum. This phenomenon is highly visible for pollutants with small particles, such as cement, red soil and ash. Regardless of the variation in reflectivity, contrast appears and therefore the detection of dust is feasible, as demonstrated when using the machine learning model. The machine learning model is very simple as it only distinguishes a polluted from a clean reflector. Much more data would be necessary to detect the concentration and the type of pollutant. However, this model demonstrates the ability of the proposed procedure to detected polluted reflectors in automated procedures. Further research in this area would be necessary to design efficient fully automated methods.

The conclusions of this work can be extrapolated to natural conditions with natural pollutants. Thus, helping develop methods to increase the efficiency of periodic cleaning maintenance operations. Nevertheless, the experiment procedure presents limitations in relation to the actual application on reflectors installed in real solar plants as the pollutant density can greatly vary depending on natural rainfall, wind, humidity and temperature. Moreover, the type of pollutant and the mixture of different pollutants play a crucial role in the degradation of the panels, where pollutants with small particles always generate greater impact.

## Figures and Tables

**Table 1:** Particle size for each pollutant.

Pollutant	Particle Size (μm)
Ash	0.5–300
Red soil	2
Cement	7–200
Limestone	500
Fine sand	1–150
Coarse sand	62.5–2000

**Table 2:** Technical specifications of the infrared camera FLIR T450sc used in the experiments.

Camera	FLIR T450sc
Temperature range	−20 to +120 ${}^{{}^{\circ}}$C
Thermal sensitivity/NETD	30 mK at 300 ${}^{{}^{\circ}}$C
Detector	320 × 240 UFPA
Pixel size	25 × 25 μm
Spectral range	7.5–13 μm
Frequency	60 Hz
Spatial resolution	1.36 mrad
Field of view (FOV)	25${}^{\circ }$ × 19${}^{\circ }$
Detector pitch (μm)	25

**Table 3:** Technical specifications of the visible camera Mikrotron EoSens MC1364 used in the experiments.

Camera	Mikrotron EoSens MC1364
Detector	1280 × 1024 UFPA
Pixel size	14 × 14 μm
Sensor type	CMOS (Complementary Metal–Oxide–Semiconductor)
Spectral range	Visible (RGB)
Frequency	81 Hz
Interface	GigE Vision
Sensitivity	10.2 V/lux*s @ 550 nm
Dynamic range (μm)	57 dB/up to 90 dB

**Table 4:** Density of the pollutants in the tests.

Weight of the Pollutant (g)	Resulting Density (g/cm${}^{2}$)
5	0.0994
2	0.0397
0.5	0.0099
0.1	0.0019

**Table 5:** Results for the reflectivity in the infrared spectrum.

Pollutant	Reflectivity							
	5 g		2 g		0.5 g		0.1 g	
	Avg	Std	Avg	Std	Avg	Std	Avg	Std
Ash	0.357	0.036	0.253	0.025	0.126	0.017	0.132	0.015
Red soil	0.376	0.031	0.284	0.026	0.133	0.030	0.124	0.011
Cement	0.380	0.021	0.306	0.013	0.115	0.023	0.105	0.034
Limestone	0.177	0.018	0.188	0.032	0.090	0.017	0.078	0.012
Coarse sand	0.128	0.032	0.218	0.030	0.150	0.031	0.164	0.029
Fine sand	0.105	0.022	0.160	0.021	0.133	0.034	0.167	0.019

**Table 6:** Relative reflectivity of the pollutants in the visible spectrum.

Pollutant	Relative Reflectivity (%)							
	5 g		2 g		0.5 g		0.1 g	
	Avg	Std	Avg	Std	Avg	Std	Avg	Std
Ash	0.00	-	0.00	-	0.00	-	11.43	19.33
Red soil	0.00	-	0.00	-	0.00	-	20.67	19.65
Cement	0.00	-	0.00	-	0.00	-	0.00	-
Limestone	0.00	-	0.00	-	48.57	30.14	45.71	31.57
Fine sand	0.00	-	0.00	-	20.00	31.81	28.57	24.57
Coarse sand	0.00	-	37.14	45.74	28.57	37.37	51.43	20.98
